# Exploring the Role of Mutations in Fanconi Anemia Genes in Hereditary Cancer Patients

**DOI:** 10.3390/cancers12040829

**Published:** 2020-03-30

**Authors:** Jesús del Valle, Paula Rofes, José Marcos Moreno-Cabrera, Adriana López-Dóriga, Sami Belhadj, Gardenia Vargas-Parra, Àlex Teulé, Raquel Cuesta, Xavier Muñoz, Olga Campos, Mónica Salinas, Rafael de Cid, Joan Brunet, Sara González, Gabriel Capellá, Marta Pineda, Lídia Feliubadaló, Conxi Lázaro

**Affiliations:** 1 Hereditary Cancer Program, Catalan Institute of Oncology, IDIBELL-IGTP-IDIBGI, 08908 Hospitalet de Llobregat, Spain; jdelvalle@iconcologia.net (J.d.V.); profes@iconcologia.net (P.R.); jmoreno@igtp.cat (J.M.M.-C.); samibelhadj@hotmail.com (S.B.); gvargas@idibell.cat (G.V.-P.); ateule@iconcologia.net (À.T.); rcuesta@iconcologia.net (R.C.); xmunoz@iconcologia.net (X.M.); ocampos@iconcologia.net (O.C.); msalinas@iconcologia.net (M.S.); Jbrunet@iconcologia.net (J.B.); sgonzalez@iconcologia.net (S.G.); gcapella@idibell.cat (G.C.); mpineda@iconcologia.net (M.P.); lfeliubadalo@iconcologia.net (L.F.); 2 Program in Molecular Mechanisms and Experimental Therapy in Oncology (Oncobell), IDIBELL, 08908 Hospitalet de Llobregat, Spain; 3 Centro de Investigación Biomédica en Red de Cáncer (CIBERONC), 28029 Madrid, Spain; 4 Oncology Data Analytics Program (ODAP), Catalan Institute of Oncology, 08908 Hospitalet de Llobregat, Spain; alguerra@iconcologia.net; 5 Consortium for Biomedical Research in Epidemiology and Public Health (CIBERESP), 28029 Madrid, Spain; 6 Medical Oncology Department, Catalan Institute of Oncology, IDIBELL, 08908 Hospitalet de Llobregat, Spain; 7 Genomes for Life—GCAT lab Group, Institut Germans Trias i Pujol (IGTP), 08916 Badalona, Spain; rdecid@igtp.cat

**Keywords:** Breast cancer risk, Breast and ovarian cancer risk, Fanconi Anemia, Hereditary Cancer, NGS panel sequencing

## Abstract

Fanconi anemia (FA) is caused by biallelic mutations in FA genes. Monoallelic mutations in five of these genes (*BRCA1, BRCA2, PALB2, BRIP1* and *RAD51C*) increase the susceptibility to breast/ovarian cancer and are used in clinical diagnostics as bona-fide hereditary cancer genes. Increasing evidence suggests that monoallelic mutations in other FA genes could predispose to tumor development, especially breast cancer. The objective of this study is to assess the mutational spectrum of 14 additional FA genes (*FANCA, FANCB, FANCC, FANCD2, FANCE, FANCF, FANCG, FANCI, FANCL, FANCM, FANCP, FANCQ, FANCR* and *FANCU*) in a cohort of hereditary cancer patients, to compare with local cancer-free controls as well as GnomAD. A total of 1021 hereditary cancer patients and 194 controls were analyzed using our next generation custom sequencing panel. We identified 35 pathogenic variants in eight genes. A significant association with the risk of breast cancer/breast and ovarian cancer was found for carriers of *FANCA* mutations (odds ratio (OR) = 3.14 95% confidence interval (CI) 1.4–6.17, *p* = 0.003). Two patients with early-onset cancer showed a pathogenic FA variant in addition to another germline mutation, suggesting a modifier role for FA variants. Our results encourage a comprehensive analysis of FA genes in larger studies to better assess their role in cancer risk.

## 1. Introduction

Fanconi anemia (FA) is a rare genetic condition originated from a DNA repair deficiency that causes a broad spectrum of clinical features of variable penetrance, mainly, progressive bone marrow failure (depending on the affected gene), congenital defects and cancer predisposition [1]. FA is usually inherited as an autosomal recessive genetic disease, although X-linked inheritance and dominant inheritance have also been described.

Hitherto, 22 genes have been described as FA genes: *FANCA, FANCB, FANCC, FANCD1/BRCA2, FANCD2, FANCE, FANCF, FANCG/XRCC9, FANCI, FANCJ/BRIP1, FANCL/PHF9, FANCM, FANCN/PALB2, FANCO/RAD51C, FANCP/SLX4, FANCQ/ERCC4, FANCR/RAD51, FANCS/BRCA1, FANCT/UBE2T, FANCU/XRCC2, FANCV/REV7* and *FANCW/RFWD3* [2]. The proteins encoded by these genes participate in the FA pathway involving DNA repair and genome maintenance processes when cell DNA damage occurs. These proteins are essential for inter-strand crosslink repair, and they also participate in homologous recombination and non-homologous end joining [3]. The *FANC-A, -B, -C, -E, -F, -G, -L* and *-M* genes encode the proteins that form the core complex, which monoubiquitinates the FANCI/FANCD complex formed by the dimer of FANCD2 and FANCI. The remaining proteins are downstream effectors in the FA pathway and their deficiency does not abolish the monoubiquitination of the I/D complex [4]. However, a recent publication described that biallelic FANCM mutations do not cause classical FA and therefore should not be considered a canonical FA gene [5], although these biallelic carriers showed risk for breast cancer, chemotherapy toxicity and may display chromosome fragility.

Apart from conditions caused by biallelic mutations in FA genes, it is well known that monoallelic mutations in certain FA genes (*BRCA1, BRCA2, BRIP1, PALB2* and *RAD51C*) are clearly related with hereditary breast and/or ovarian cancer predisposition [6], and these genes are bona-fide hereditary breast and ovarian cancer (HBOC) predisposition genes. Hence, cancer risks have been estimated for heterozygous mutations in these genes, and clinical management is also well established and accepted. However, the role of monoallelic mutations in the remaining FA genes regarding cancer predisposition is a matter of discussion. Over the last few years, several case-controls studies have indicated that monoallelic *FANCM* [7,8,9,10,11,12,13,14,15] truncating mutations are breast cancer risk factors; in addition, there are inconsistent results regarding *FANCA* [16,17,18,19], *FANCC* [20,21,22,23,24], *SLX4* [25,26,27] and *XRCC2* [28,29,30].

In the midst of these conflicting results, the use of comprehensive next generation sequencing (NGS) gene panels could shed some light on the role of FA genes in the context of hereditary cancer in general. For this reason, we analyzed these FA genes in our entire cohort of hereditary cancer patients, not just breast and ovarian cancer. Our I2HCP panel [31] contains, besides the five bona-fide HBOC genes, the following 14 FA genes: *FANCA, FANCB, FANCC, FANCD2, FANCE, FANCF, FANCG/XRCC9, FANCI, FANCL/PHF9, FANCM, FANCP/SLX4, FANCQ/ERCC4, FANCR/RAD51* and *FANCU/XRCC2*. Here, we present the mutation profile of these 14 genes in our cohort of 1021 hereditary cancer patients and compare it with the mutational spectrum found in a control population consisting of 194 cancer-free individuals from our region as well as the GnomAD (genome aggregation database) non-cancer, European non-Finnish cohort.

## 2. Results

A prospective cohort of 1021 unrelated cancer cases with clinical suspicion of hereditary cancer was screened for mutations in the following 14 FA genes: *FANCA, FANCB, FANCC, FANCD2, FANCE, FANCF, FANCG/XRCC9, FANCI, FANCL/PHF9, FANCM, FANCP/SLX4, FANCQ/ERCC4, FANCR/RAD51* and *FANCU/XRCC2*. The sequence of all coding regions and exon–intron boundaries (±20) was obtained by NGS and was also used to determine putative copy number variations (CNVs), which were validated by MLPA analysis. Other pathogenic variants identified in the clinical testing workflow, according to the clinical cascades presented in Feliubadaló et al. [32], are depicted in Table S1.

**Figure 1:**
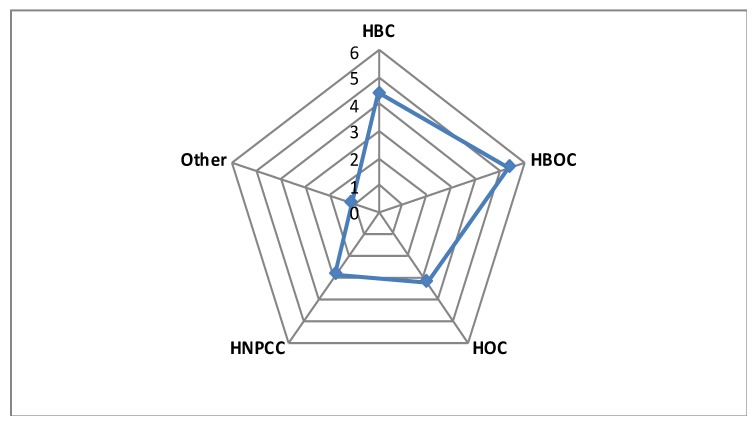
The diagram represents the percentage of pathogenic variants in the 14 Fanconi anemia (FA) genes analyzed per clinical suspicion group. HBC: Hereditary Breast Cancer Patients; HOC: Hereditary Ovarian Cancer Patients; HBOC: Hereditary Breast and Ovarian Cancer Patients; HNPCC: Hereditary non-polyposis colorectal cancer.

Our study identified 35 heterozygous carriers of 22 pathogenic/likely pathogenic variants in the patient cohort. The most frequently mutated genes were *FANCA, FANCL* and *FANCM*, whereas no mutation was identified in *FANCB, FANCD2, FANCF, FANCG, SLX4, ERCC4* and *XRCC2* (Table 1). Six mutations were identified in our set of 194 healthy controls. Overall, a monoallelic mutation in a FA gene was identified in 3.4% of patients in our hereditary cancer cohort, a percentage very similar to that identified in the control cohort studied here (3.1%). However, distribution of mutations by clinical phenotype evidenced that pathogenic variants were mainly present in patients with a history of breast cancer, or breast and ovarian cancer. The percentage of pathogenic mutations increased to 4.6% (counting only women) in cases with breast cancer, being higher (5.5% counting only women) in those with breast and ovarian cancer (Figure 1).

Details of all identified mutations and the clinical characteristics of the carriers are depicted in Table S2. Intriguingly, in three cases, an additional mutation in a hereditary cancer gene was also identified. Two of them were carriers of a deleterious variant in *FANCA*, one corresponds to a female with breast cancer at age 35 (patient ID 19136 in Table S2), carrier of a pathogenic variant in *ATM* and the other was diagnosed with ovarian cancer at age 49 and also harbors a mutation in *SDHB* (patient ID 6988 in Table S2). The third case, with a deleterious mutation in *FANCL*, is a Lynch syndrome patient with a mutation in *MLH1* who suffered colorectal cancer at age 29 (patient ID 19012 in Table S2). Interestingly, six of the mutations were identified in more than one individual, *p*.(Thr372Asnfs*13) in *FANCL* was identified in 10 individuals and *p*.(Arg1931*) in *FANCM* in 3 individuals, the remaining were identified in two cases each (Table S2).

DECoN (Detection of Exon Copy Number) analysis of NGS data identified 14 putative CNVs in the patient cohort that were validated by MLPA. Two turned out to be true positives consisting of a deletion of exons 6–13 in *FANCL* and a deletion of exons 11–37 in *FANCA*. Furthermore, 1605 variants of unknown significance (VUS) were identified in both cohorts, 589 unique (Table S3). Some of these VUS were predicted, by multiple in-silico tools, to alter correct splicing. Among them we were able to obtain lymphocytes for RNA analysis in five patients harboring the following mutations: *FANCA:* c.523-25_523-20delTTGTTT, c.576C > T, c.2217G > A and c.2602-9_2602-8delCT and *FANCM:* c.4222 + 5G > A. RNA analysis of these five variants did not identify any aberrant transcript (data not shown), so they remained classified as VUS.

Lastly, we compared the mutational profile of our cohort of patients with data from the European (non-Finnish, non-cancer) GnomAD 2.1 population. After the first analysis, a possible association was only found with breast and ovarian cancer, we stratified the different populations by gender, counting only women (analysis without this stratification is shown in Table S4). By this means, only *FANCA* mutations showed a statistically significant association with an increased cancer risk (Table 1) in the combined group of hereditary breast cancer (HBC) and HBOC (odds ratio (OR) = 3.14 (95% confidence interval (CI) 1.4–6.17) *p* = 0.003). However, this association must be taken with caution since 3% of our in-house control cohort (from GCAT, Genomes for Life Cohort) carried deleterious *FANCA* mutations compared with 0.6% of the European non-Finnish cohort, being 0.98% in our complete cohort of hereditary cancer patients. 

## 3. Discussion

In this study, we have evaluated the presence of deleterious mutations in 14 FA genes in a cohort of 1021 patients in the context of hereditary cancer. In total, 3.4% of the patients have a pathogenic variant in one of these genes. This percentage is higher in the group of women patients with breast cancer (4.4%) and increases in the group of women patients with a history of breast and ovarian cancer (5.4%). We analyzed these genes in two European populations, a general adult population cohort from Spain (GCAT) and in the European non-Finnish GnomAD cohort, identifying pathogenic variants in 3.1% and 3.0% of control individuals, respectively. If only women are considered, the percentages increase to 5% in GCAT and 3.2% in GnomAD. The NGS analysis performed allowed us not only to detect single nucleotide variants but also to screen for CNVs. By this means, we identified two large intragenic deletions in *FANCL* and *FANCA*, highlighting the importance of searching for this type of variant when analyzing FA genes in patients with Fanconi anemia.

In general, the genes most frequently mutated in our cohort of patients were *FANCA* (*n* = 10), *FANCL* (*n* = 10) and *FANCM* (*n* = 7). Few cases were identified with mutations in *FANCI, FANCE, FANCC* (*n* = 2, in each gene) and *FANCF* and *RAD51* (*n* = 1, in each gene). No mutations were identified in *FANCB, FANCD2, FANCF, FANCG, ERCC4* and *XRCC2*, and only one pathogenic variant was identified in *SLX4*, but in a sample corresponding to a healthy control. Hence, it seems that most of these 14 FA genes do not play a major role in hereditary cancer, although our data cannot discard their relation with rare cancer syndromes or their role as modifier genes. To assess these possibilities, larger cohorts of patients with different tumor types and the use of polygenic risk score methodologies should be applied.

It is worth mentioning that one of the most frequently mutated genes in our series, as well as in the European non-Finnish GnomAD cohort, is *FANCL.* This fact is due to the high number of patients carrying the c.1111_1114dup mutation. This alteration, located in the last exon of the gene, produces a frameshift that lengthens the protein by three amino acids more than wild-type. This mutation has been described in a patient with FA, a compound heterozygote with another *FANCL* mutation [33]. Functional analysis of this mutation identified a partial correction of G2/M cell cycle arrest that results in an intermediate phenotype compatible with a hypomorphic mutation. So, the contribution to cancer risk of this variant in monoallelic carriers could be very limited but deserves further study. In our series, we also detected an enrichment of the c.5791C > T variant in *FANCM*. This alteration is the most common pathogenic *FANCM* variant in Southern Europe [34] and was associated with estrogen receptorER-negative breast cancer risk (OR = 1.96; *p* = 0.006) in a large case-control study with more than 50,000 cases and controls [15]. However, in the present study, we could not find a significant association with breast cancer risk (odds ratio = 1.46 (95% confidence interval 0.3–4.8) *p* = 0.467).

## 4. Materials and Methods

### 4.1. Patients and Controls

A total of 1021 hereditary cancer-suspected index cases, referred through our genetic counselling units, that underwent NGS panel testing based on clinical suspicion [32], were included in this study (Table 2). Genetic counselors followed international guidelines to request germline genetic tests under the suspicion of a hereditary cancer syndrome. Informed written consent for both diagnostics and research purposes was obtained from all patients included in the study and the study protocol was approved by the Ethics Committee of IDIBELL (Bellvitge Biomedical Research Institute, PR278/19).

GCAT (Cohort Study of the Genomes of Catalonia Study) is a biomedical research project designed for the study of genetic, epigenetic and environmental factors that lead to the appearance of different complex inheritance diseases in the general population [35]. Briefly, the subjects of the present study are part of the GCAT project, a prospective study that includes a cohort of a total of 19,267 participants recruited from the general population of Catalonia, a western Mediterranean region in the Northeast of Spain. All are cancer-free general population volunteers between 40 and 65 years of age. All eligible participants signed an informed consent agreement form. The GCAT study was approved by the local ethics committee (IRB00002131) (Germans Trias University Hospital) in 2013.

### 4.2. DNA Isolation

Genomic DNA was extracted from peripheral blood lymphocytes using the FlexiGene DNA Kit (Qiagen GmbH, Hilden, Germany) in the patient cohort and the ReliaPrep DNA Kit (Promega, Wisconsin, USA) in the GCAT cohort.

### 4.3. NGS Panel Testing

All patients and controls were analyzed by our validated custom NGS panel I2HCP, which comprises 122–135 hereditary cancer-associated genes, depending on the version used [31]. This panel includes the *FANCA, FANCB, FANCC, FANCD2, FANCE, FANCF, FANCG/XRCC9, FANCI, FANCL/PHF9, FANCM, FANCP/SLX4, FANCQ/ERCC4, FANCR/RAD51* and *FANCU/XRCC2* genes. Library preparation methods and bioinformatics pipeline were described previously [31]. The regions of interest analyzed include all coding regions and ±20 nucleotides intron/exon boundaries. For this study we considered as a pathogenic or likely pathogenic variant (pathogenic variant hereinafter) mutations that originate a premature stop codon, missense variants described in the literature as clearly pathogenic in FA patients and mutations affecting canonical splice site positions (+1, +2, −1,−2). All pathogenic variants were confirmed by Sanger sequencing.

Copy number analysis was performed from NGS data using the DECoN [36] tool with parameter optimization for our panel (Moreno et al., submitted manuscript). However, the *FANCB* gene was not included in this analysis as it is located on the X chromosome, which greatly complicates the identification of CNVs with our pipeline. Likewise, *FANCD2* was also excluded from this analysis due to the presence of pseudogenes, which generate false positives in both directions (deletions and duplications). For the rest of the genes, we used the Bayesian-factor value, which is a good predictor of the reliability of the DECoN’s result to select the most likely true positive copy number alterations to be confirmed. All samples with a suspicion of alteration were subsequently analyzed by MLPA using custom probes according to the instructions provided by MRC-Holland in order to validate or discard the presence of CNVs (https://support.mlpa.com/downloads/files/designing-synthetic-mlpa-probes).

### 4.4. RNA Analysis

Lymphocytes were isolated by centrifugation of peripheral blood samples from carriers and controls. Cells were cultured in PB-Max medium for 5 to 7 days and treated with puromycin 4 to 6 h before RNA extraction in order to prevent the potential degradation of unstable transcripts by nonsense-mediated decay (NMD). Total RNA was isolated using TRIzol reagent according to the manufacturer’s instructions. One microgram of total RNA was reverse transcribed using the iScript cDNA Synthesis kit (Bio-Rad Laboratories, Hercules, CA, USA). cDNA amplification was performed with specific primers that encompassed the region of interest. Transcriptional profiles from carriers were compared to those derived from control lymphocytes cultures, both by agarose gel analysis and Sanger sequencing. Primer sequences and PCR conditions are available upon request.

### 4.5. GnomAD Analysis

The GnomAD non-Finnish European, non-cancer subpopulation (Genome Aggregation Database, v2.1.1, http://gnomad.broadinstitute.org/) was used as a control population. Variants were exported and filtered to identify predicted loss of function variants in FA genes.

### 4.6. Statistical Analysis

Differences in allele frequency between cases and controls were determined by the Fisher exact test. Odds ratios (OR) and the corresponding 95% confidence intervals (CI) were determined for two by two comparisons. Statistical tests were carried out using R v.3.5.1. (R Foundation for Statistical Computing, Vienna, Austria).

## 5. Conclusions

Our study identified an increased number of pathogenic mutations in *FANCA* in the HBC/HBOC group (*p* = 0.003). In addition, we observed a higher number of mutations in the remaining genes (5.4% versus 3.2%) in the group of patients with a history of breast and ovarian cancer. Two out of the three cases with additional mutations in other moderate/high-penetrance genes, had been diagnosed with cancer at a very young age, suggesting a modifier role for FA mutations. Altogether, our results encourage further studies in larger cohorts to assess the role and risks of deleterious variants in these genes to determine their potential future use in clinical settings.

## Figures and Tables

**Table 1:** Summary of (Likely) Pathogenic Variants in 14 FA genes in the different clinical groups (only women are counted).

		Clinical Suspicion					GCAT Women Cohort (*n* = 100)	GnomAD European >23,000 women ^β^	Study Cohort Versus NFE ^γ^, Non-Cancer GnomAD (OR/95%CI/*p*-Value)		
Genes	Pathogenic Variants	Breast (HBC)	Ovary (HOC)	Breast + Ovary (HBOC)	HNPCC ^α^	Other			All Patients	HBC + HOC + HBOC	HBC + HBOC
***FANCA***	10	7	0	2	1	0	3	147	1.94/0.91–3.7/0.047	2.34/1.04–4.59/0.02	3.14/1.4–6.17/0.003*
***FANCL***	8	3	1	3	1	0	1	187	1.22/0.52–2.46/0.549	1.42/0.56–3/0.356	1.63/0.59–3.64/0.283
***FANCM***	6	2	3	0	1	0	0	159	1.07/0.38–2.39/0.828	1.19/0.38–2.85/0.618	0.63/0.08–2.34/0.774
***FANCI***	1	1	0	0	0	0	0	25	1.14/0.03–7/0.593	1.12/0,04–9.29/0.492	2.02/0.05–12.4/0.399
***FANCE***	2	1	0	0	1	0	0	17	1.14/0.03–6.97/0.593	1.52/0.04–9.3/0.492	2.03/0.05–12.4/0.399
***FANCC***	2	1	1	0	0	0	0	44	1.29/0.15–4.97/0.67	1.72/0.2–6.63/0.332	1.15/0.03–6.78/0.586
***FANCF***	1	1	0	0	0	0	0	26	1.09/0.02–6.6/0.608	1.45/0.04–8.88/0.506	1.94/0.05–11.87/0.412
***RAD51***	1	1	0	0	0	0	0	4	7.12/0.14–72/0.159	9.49/0.19–96/0.122	12.7/ 0.26–128/0.093
***SLX4***	0	0	0	0	0	0	1	36	NA	NA	NA
***ERCC4***	0	0	0	0	0	0	0	22	NA	NA	NA
***FANCB***	0	0	0	0	0	0	0	0	NA	NA	NA
***FANCD2***	0	0	0	0	0	0	0	21	NA	NA	NA
***FANCG***	0	0	0	0	0	0	0	43	NA	NA	NA
***XRCC2***	0	0	0	0	0	0	0	22	NA	NA	NA
**TOTAL**	31	17	5	5	4	0	5	753			

α Hereditary non-polyposis colorectal cancer; ^β^ The number of GnomAD non-Finnish, non-cancer women is slightly variable per gene but in all cases was greater than 23,000 γ NFE: non-Finnish European.

**Table 2:** Summary of the hereditary cancer cohort by clinical suspicion.

Clinical Suspicion	Number of Patients (Women)
Hereditary breast cancer, HBC	385 (370)
Hereditary non-polyposis colon cancer, HNPCC	210 (130)
Hereditary ovarian cancer, HOC	154 (154)
Other hereditary cancer conditions	102 (55)
Hereditary breast and ovarian cancer, HBOC	93 (90)
Familial (attenuated) adenomatous polyposis, FAP/AFAP	77 (19)
Total	1021 (818)

A set of 194 cancer-free controls (100 women) from GCAT, Genomes for Life Cohort, was also analyzed.

## Supplementary Materials

The following are available online at https://www.mdpi.com/2072-6694/12/4/829/s1: Table S1: List of pathogenic variants in other hereditary cancer genes, Table S2: List of (likely) pathogenic variants with clinical detail of the patients’ tumors and family cancer history, Table S3: List of VUS variants identified, Table S4: Summary of (Likely) Pathogenic variants in 14 FA genes in the different groups without gender stratification.
